# The Absence of Close Social Network Connections: A Case Study Investigation in Social Isolation

**DOI:** 10.1093/geroni/igaa057.550

**Published:** 2020-12-16

**Authors:** Raeann LeBlanc, Rachel Walker

**Affiliations:** 1 University of Massachusetts, Amherst, Amherst, Massachusetts, United States; 2 University of Massachusetts Amherst, Amherst, Massachusetts, United States

## Abstract

Social isolation is an emerging phenomenon known to significantly influence health outcomes and carries specific risk in older age. As part of a larger study exploring the social network effects on health among persons age 65 and above, it was found that four cases from a larger sample (n=89) could not name any one from their social relationships supportive to them in managing their health while living with complex chronic health needs. In addressing these findings, these cases, bounded spatially by individual social networks and temporally by the time of the study interview, served the basis for intensive analysis using multiple data points from in-depth interviews and survey measures. Measures included descriptive data (social networks, demographics) and measures of health (SF-12), and social support (MOS-SSS). Each participant case identified as female gender and single, three lived alone, had a high burden of chronic conditions and poor health. All had recent hospitalizations (1 or more within the past year). The structures of their social networks varied in type of relationship roles and size. Social support was perceived overall as low. This study offers a specific contribution to research on social connection/isolation. This phenomenon is relative to specific contexts. Findings emphasize that social isolation can be specific to certain aspects of identity and poorer health in older age. Additional research on the functions and qualities of social networks, in addition to the structure, are important to specify in future research and knowledge development for practice assessment to determine social connection and isolation.

